# Bapoma case report: BRCA1-associated protein–inactivated melanocytic tumor as first presenting symptom of BRCA1-associated protein tumor predisposition syndrome in a pediatric patient

**DOI:** 10.1016/j.jdcr.2025.10.073

**Published:** 2025-11-21

**Authors:** Acacia Bowden, Leah Laageide, Glynis Scott, Marc Brown, Kevin Vo, Jeffrey R. Andolina, Jinia El-Feghaly

**Affiliations:** a Department of Pediatrics, Golisano Children’s Hospital, Rochester, New York; b Department of Dermatology, University of Rochester Medical Center, Rochester, New York

**Keywords:** BAP1, Bapoma, BIMT, genetic testing, pediatric dermatology, tumor predisposition syndrome

## Introduction

Located on chromosome 3p21, BRCA1-associated protein (BAP1) encodes a ubiquitin carboxy-terminal hydroxylase suppressor protein that binds the BRCA1 (BReast CAncer gene 1) protein, increasing BRCA1-mediated tumor suppression while also contributing to DNA damage repair, cell cycle regulation, and growth.^1^ Germline mutations of *BAP1* induce both malignant and benign tumors of the eyes, skin, kidneys, and mesothelium that collectively define BRCA-1 associated protein (BAP1) tumor predisposition syndrome (BAP1-TPDS), first described in the early 2000s and inherited in an autosomal dominant manner.^2^^,^^3^

BAP1-inactivated melanocytic tumors (BIMTs) are observed in approximately 75% of BAP1-TPDS patients and may arise by the second decade of life.^4^ Screening tests, including annual skin and ophthalmic examinations, are recommended in early adulthood.^5^ *BAP1* mutations may also be somatic; however, their role in BAP1-TPDS is not fully elucidated. Ewens et al (2018) recently identified patients with uveal melanoma reporting that positive somatic *BAP1* mutations also had significantly higher risk of metastasis than tumors with germline or no mutations.^1^

We report the clinical course of a pediatric patient with a changing variegated plaque on the trunk and an extensive family history of skin cancer with subsequent biopsy-proven and genetically confirmed BAP1-TPDS.

## Case report

**Fig 1:**
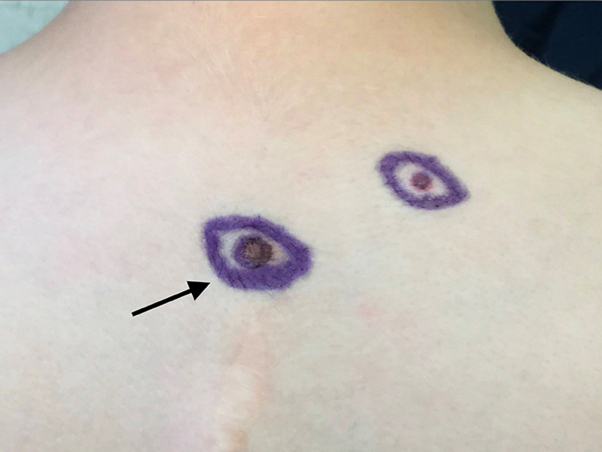
Lower left lesion: BAPoma presenting as a 1 × 0.8 cm dark brown papule with mild surrounding erythema (indicated with the *black arrowhead*). Upper right lesion: planned re-excision to check the margins from a previous biopsy showing a dysplastic nevus; no residual nevus was identified.

**Fig 2:**
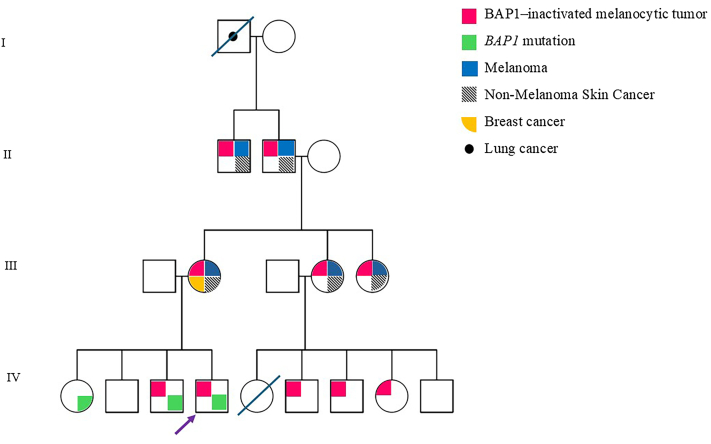
Pedigree. The proband (indicated with the *purple arrowhead*) presented with a BAP1-inactivated melanocytic tumor at 9 years old. Filled-in colored symbols indicate family members affected by BAP1-inactivated melanocytic tumors, BAP1 mutation, and/or cancer. *BAP1*, BRCA1-associated protein.

**Fig 3:**
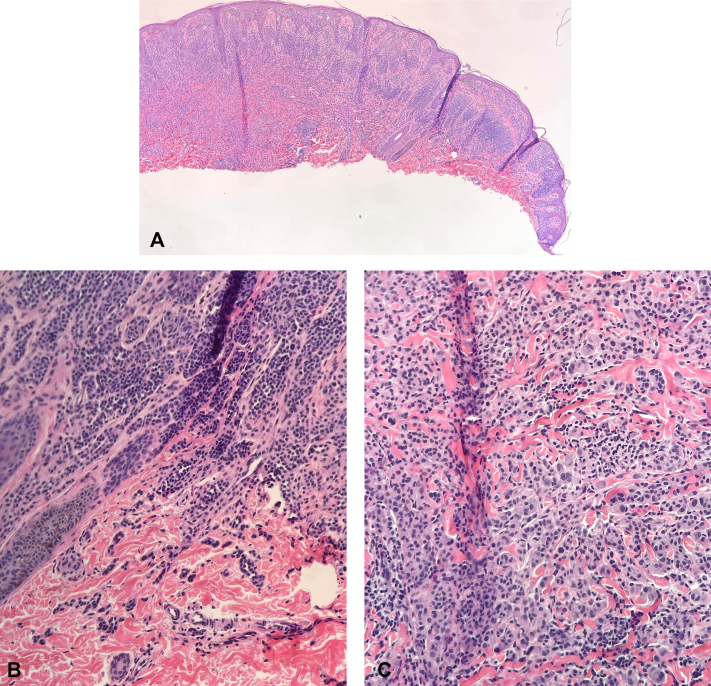
Histopathology: H&E showing compound melanocytic proliferation with combined features of a dysplastic, congenital, and Spitz nevus, including a mixed population of large, epithelioid atypical cells and smaller unremarkable dermal lymphocytoid melanocytes. **A,** 40× magnification: low power overview with enlarged melanocytes lacking maturation in the center and the more conventional nevus component surrounding it. **B,** 100× magnification: higher magnification of the conventional melanocyte component, with deeper melanocytes exhibiting maturation. **C,** 100× magnification: higher power view of the “atypical” component, with the deeper melanocytes retaining abundant cytoplasm and large nuclei even in the mid-to-deep dermis. *H&E*, Hematoxylin and eosin.

**Fig 4:**
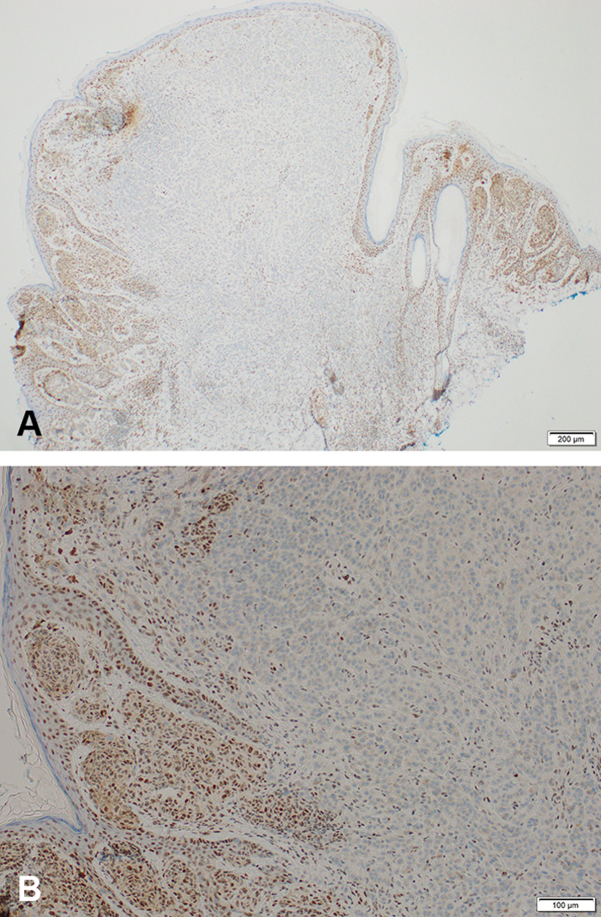
Immunocytochemical stains at **(A)** 4× magnification and **(B)** 10× magnification showing loss of BAP1 in the type A (epithelioid) melanocytes with retention in the type B (lymphocytoid) dermal melanocytes. *BAP1*, BRCA1-associated protein.

A previously healthy 9-year-old male presented to dermatology for a brown lesion on the right upper back. The lesion was noticed a year ago but had developed a new red-pink discoloration centrally over the last 3 to 4 months. Skin examination revealed a 1 × 0.8 cm dark brown papule with mild surrounding erythema (Fig 1). Family history was notable for several melanoma and nonmelanoma skin cancers. The patient’s mother, 2 maternal aunts, grandfather, and great uncle had documented melanomas in addition to cutaneous squamous and basal cell carcinomas. His mother also had a history of stage 2 breast cancer. Other reported malignancies included lung cancer (type unknown) in his maternal great grandfather (nonsmoker) and ovarian cancer in his paternal great grandmother (Fig 2). Based on the clinical picture and the extensive family history of skin cancer, a shave biopsy was performed. Histopathology demonstrated a compound melanocytic proliferation with combined features of a dysplastic, congenital, and Spitz nevus, including a mixed population of large, epithelioid atypical cells and smaller unremarkable melanocytes (Fig 3). Immunohistochemical staining identified islands of melanocytes lacking *BAP1* expression (Fig 4). A diagnosis of a BIMT, also known as a BAPoma, was made. The patient underwent wide local excision with clear 5-mm margins. Patient is now 5 years postoperative procedure; no recurrence, new concerning lesions, or other medical comorbidities including features of BIMT have been identified during his annual visits with dermatology.

In the setting of his rare BIMT diagnosis and the family history as above, the patient was referred to pediatric hematology and oncology for evaluation for a malignancy-predisposition syndrome. Accordingly, genetic testing for BAP1-tumor predisposition syndrome was pursued in the patient and his 3 full siblings who were of ages 14, 16, and 18 years at that time. Genetic testing revealed a *BAP1*-germline pathogenic variant in the patient, his sister, and one of the 2 brothers (Fig 2). Skin examination was recommended for both siblings: the sister has undergone several biopsies and remains without any known skin cancers or BAP1-inactivated melanocytic nevi, while the affected brother underwent a single skin biopsy which confirmed a *BAP1*-inactivated melanocytic nevus. No other family members have undergone genetic testing.

## Discussion

We present a 9-year-old male patient with a biopsy-proven BIMT and genetically confirmed BAP1-TPDS. To our knowledge, this is one of the youngest reported cases of BAP1-TDS in a pediatric patient with cutaneous findings. Literature cites a 16-year-old with a uveal melanoma in the setting of a germline *BAP1* mutation. This case highlights the importance of diagnostic surveillance of this cancer predisposition syndrome in pediatric patients with BIMT, particularly if concomitant family history of cutaneous and visceral malignancies is present.

*BAP1* is a tumor suppressor gene with germline mutations associated with several tumors: uveal and cutaneous melanoma, pleural and peritoneal mesothelioma, renal cell carcinoma (RCC), lung adenocarcinoma, and meningioma. Predisposition to these tumors is collectively part of the autosomal dominant BAP1-tumor predisposition syndrome.^5^ The phenotype of BAP1-TPDS is still evolving, but the following are confirmed BAP1-TPDS tumors in order of decreasing likelihood: BIMT, formerly “atypical Spitz tumors,” uveal melanoma, malignant mesothelioma, cutaneous melanoma, RCC, and basal cell carcinoma. Hepatocellular carcinoma, cholangiocarcinoma, and meningioma are rare manifestations of BAP1-TPDS. Other cancers with limited relation to BAP1-TDS include breast cancer, neuroendocrine tumors, nonsmall cell lung adenocarcinoma, thyroid cancer, and urinary bladder cancer.^6^ However, there is a reported case of a BAPoma being diagnosed in the setting of a *BAP1* germline mutation and family history of breast cancer.^7^

Although the prevalence of BAP1-TPDS remains unknown, the Genome Aggregation Database reports a carrier frequency of 1:26,837 in the general population.^6^ Penetrance of *BAP1* germline pathologic variants is also reported to be 85%.^8^ This may, however, represent an overestimation via selection bias as individuals undergoing genetic screening often carry additional malignancies.^8^ Current screening recommendations by Star et al for patients with a BAP1-TPDS core malignancy include annual dilated eye examinations for patients aged between 16 and 30 years and then every 2 years to screen for uveal melanoma. Screening for cutaneous melanoma twice a year is also recommended starting at age 18 and surveillance every 2 years for malignant mesothelioma and RCC starting at age 30.^9^^,^^10^ Recommendations are garnered from reported ages of BAP1-TPDS with BAP1-TPDS core malignancies of 16, 25, 34, and 36 for uveal melanoma, cutaneous melanoma, malignant mesothelioma, and RCC, respectively.^10^ However, pediatricians and dermatologists should be cognizant of BIMT occurring in pediatric patients, including the first decade of life, although the average age of BIMT diagnosis is 36.9 years. Per clinical and dermatoscopic examination, dome-shaped papules or nodules with pink-to-tan structureless areas, and peripheral irregular dots or globules, should raise suspicion for BIMT. Based on our findings, we suggest baseline dermatologic screening around puberty (9-12 years of age) for pediatric patients with a first-degree relative affected by melanoma, BIMT, or BAP1-TPDS, accompanied by appropriate counseling and anticipatory guidance. New or evolving lesions with atypical features should be evaluated by dermatology, with biopsy performed as indicated for histopathologic diagnosis.

Diagnostic confirmation of BAP1-TPDS involves the identification of a heterozygous germline pathogenic variant in *BAP1* via molecular genetic testing of the proband. Current approaches include single-gene testing or multigene panels. If positive for a germline *BAP1* mutation, surveillance may be considered for the proband and for family members with a *BAP1* pathogenic variant.^6^ There are currently no treatments specifically targeting patients with BAP1-TPDS; all treatment options are currently based on existing clinical guidelines for each respective cancer.^6^ We hereby report one of the youngest pediatric patients diagnosed with a BIMT leading to a BAP1-TPDS diagnosis. Accordingly, dermatologists should consider skin biopsy, genetic testing, and further diagnostic screening when BIMT and BAP1-TPDS are suspected, even in the pediatric population.

## Conflicts of interest

None disclosed.
